# Anxiety and Depression Among Health Sciences Students in Home Quarantine During the COVID-19 Pandemic in Selected Provinces of Nepal

**DOI:** 10.3389/fpubh.2021.580561

**Published:** 2021-03-01

**Authors:** Rajesh Kumar Yadav, Sushila Baral, Elina Khatri, Sony Pandey, Pawan Pandeya, Roshan Neupane, Dipendra Kumar Yadav, Sujan Babu Marahatta, Hari Prasad Kaphle, Jiwan Kumar Poudyal, Chiranjivi Adhikari

**Affiliations:** ^1^School of Health and Allied Sciences, Pokhara University, Pokhara, Nepal; ^2^Health Research Together Initiative-Nepal (HeaRT: Initiative), Kathmandu, Nepal; ^3^Monitoring and Evaluation Officer (WHP-V), Aasaman Nepal, Janakpurdham, Nepal; ^4^Department of Public Health, Manmohan Memorial Institute of Health Sciences, Kathmandu, Nepal; ^5^School of Public Health, Patan Academy of Health Sciences, Lalitpur, Nepal; ^6^Faculty of Social Sciences, Oslo Metropolitan University, Oslo, Norway; ^7^Faculty of Health Science and Technology, Nepal Open University, Lalitpur, Nepal; ^8^Department of Public Health, Shree Medical and Technical College, Chitwan, Nepal

**Keywords:** anxiety, depression, health science students, Nepal, COVID-19 pandemic

## Abstract

**Aim:** This study aimed to assess anxiety and depression among health sciences students at home quarantine during the COVID-19 pandemic in selected provinces of Nepal.

**Methods:** A web-based cross-sectional study was conducted among 409 health science students enrolled at graduate and post-graduate levels in selected universities and their affiliated colleges. Students from selected colleges were asked to fill out a survey, that was made available through email and social media outlets such as Facebook and Viber. The data were downloaded in Excel and imported to SPSS version 16 for analysis.

**Results :** The prevalence of anxiety and depression was 15.7 and 10.7%, respectively. The study showed significant associations between (i) place of province and anxiety; (ii) sleep per day and depression; (iii) hours spent on the internet per day for education and depression; (iv) postponement of final exams and depression. There were no significant associations with the socio-demographic variables.

**Conclusion:** Anxiety and depression in health science students showed correlation with the province, internet use for education, and postponement of exams. These correlations could be common among students in other fields as well. A large-scale study covering a wider geographical area and various fields of education is necessary to further evaluate the impact of COVID-19 on (health sciences) students. The integration of mental health programs both as an intervention and a curriculum level among students is critical to ensure the health of the students.

## Introduction

COVID-19 is caused by the 2019 novel coronavirus or 2019-nCoV (1). This virus is in the same family of viruses as SARS. COVID-19 caused by the SARS-CoV-2 was first detected in Wuhan China in late December 2019 and has since spread all over the world (2). On January 30, 2020, the World Health Organization (WHO) declared the outbreak a public health emergency of international concern as the number of cases began to escalate across the globe (3).

The WHO declared the COVID-19 outbreak as a pandemic on March 11, 2020, when the confirmed cases reached 118,319 with 4,922 deaths worldwide (4). By May 18, 2020, there were 4,825,902 confirmed cases of COVID-19, with 317,101 deaths worldwide. According to the WHO, the case fatality rate was estimated to be around 2%. However, a few reports suggested that the rate ranged from 0.3 to 0.6% (5).

The COVID-19 pandemic has had a profound and pervasive impact on global mental health (6). It was reported that nearly all people affected by or during such global emergencies will experience some level of psychological distress, which for most will improve over time. The prevalence of mental disorders is expected to double compared to an emergency (7). The COVID-19 pandemic brought not only the risk of death from infection but also unbearable psychological pressure (8). A study done in French Universities showed that mental health-related quality of life was poorer than physical health (*p* < 0.0001) among health science students (9).

In Nepal, the first case of COVID-19 was reported on January 23, 2020 and the second case on March 23, 2020 (10). The government of Nepal announced a countrywide lockdown when the second case was announced (11, 12). Along with suggested physical distancing, people are also maintaining a certain social distance from friends and families. In addition, COVID-19 has been heavily stigmatized and can inevitably accelerate anxiety and depression (13). These circumstances have affected students in unprecedented ways because students generally have plans and ambitions for their future. Students faced an enormous disruption to their lives and education which has added a layer of uncertainty in their future. This has been a new and challenging context for students.

Health sciences students include those studying in the field of medicine and paramedics. Most of them have practical classes, field-work duties, and dates of graduation that have been postponed due to the pandemic. Many are instead taking classes online which may not be satisfactory and effective. A recent study on anxiety and depression during the COVID-19 pandemic among medical students in Nepal showed that a significant number of medical students were suffering from high levels of anxiety and depression. About 11.8 and 5.5% of medical students suffer from anxiety and depression, respectively (14). Another study found that 20.4% of students had a moderate level of anxiety, 6.6% had severe anxiety, and 2.8% had extreme levels of anxiety. Gender, age, level of education, and living arrangements were significantly associated with higher levels of anxiety (15). A study on the impacts of COVID-19 on college student's mental health in the United States showed that around 71% had increased stress and anxiety due to the COVID-19 outbreak. Multiple stressors were identified that contributed to increased levels of stress, anxiety, and depressive thoughts (16).

Most of the graduate and post-graduate level health science students are studying in either a semester or annual system. When the WHO declared the COVID-19 outbreak as a pandemic, many students were preparing to take final examinations (their exam schedules had been published) but a sudden announcement of complete lockdown by the government shattered the expectations of students. With the lockdown, universities were closed and exams were postponed. There was, and still is, a dilemma regarding the resumption of the academic activities. Amidst the uncertainty of all academic activities, and virtual classes; students suffer more from the separation and being physical/socially distanced from the college environment and thus are more likely to develop depression and anxiety.

Assessing the level of anxiety and depression among health science students showed the existence of health problems. A French online survey has shown 52.6% anxiety and 11.6% depression among health science students (9). It is critical to understand the mental health status of health science students and possible ways to mitigate such problems to protect them from anxiety and depression. The main objective of this study was to assess anxiety, depression, and associated factors that will guide interventions to maintain psychological well-being.

## Materials and Methods

### Study Design and Setting

A web-based cross-sectional study was conducted among health science students studying in different provinces of Nepal. Graduate and post-graduate level students of selected colleges were invited to enroll in the study. Since the study design was web-based, only students who had internet access were able to fill out the online form.

### Sample and Sampling

Health science students studying at graduate and postgraduate levels, aged 8 years and over were the most important selection criteria for participants. The Cochran formula was used to calculate the sample size (*n* = Z^2^pq/d^2^). Based on the study by Kunwar et al. prevalence of depression among health science students during COVID-19 was (*p*) = 0.41 (17) and the maximum allowable error was calculated to be (*d*) = 5%. The sample size was 372. Adding a 10% non-response rate, the final sample size for the study was 409. Colleges from different provinces were representatively selected. Altogether nine colleges were selected, among which one college from province 1 and province 2; three colleges from province 3; two colleges from province 4 and one college from province 5 and 6 was selected. A list of students of selected colleges was obtained from the head of the department; students were chosen proportionately to reach the sample for this study. In addition to coordination with the course manager, the selected students were reminded three times a week, during their online classes.

### Data Collection

Anxiety and depression are influenced by socio-demographic factors. We used a structured questionnaire that has five parts: socio-demographic characteristics, educational factors, health-related factors, technological factors, anxiety (seven items), and depression (nine items). Data collection was done within 1 week in the 1st week of June 2020. Around 6–10 min was enough to fill in the questionnaire. They were regularly monitored to ensure they had access to the forms and submitted forms were checked for completeness. We followed-up with students who were disturbed due to internet issues to ensure their participation and that they completed the form.

### Socio-Demographic Characteristics

Age, sex, religion, ethnicity, place of residence, living arrangements (own house/rent) were collected.

### Education Factors

Educational data including the level of education, education systems, attendance of virtual classes, frequency and duration of virtual classes, pending assignments, postponement of the exam, time spent on study, and internet-related data were collected.

### Health-Related Factors and Technological Factors

Diet pattern, exercise, source of internet and its strength, the device used for internet, technical ability to operate applications and related data were collected.

### Generalized Anxiety Disorder Assessment (GAD-7)

Generalized Anxiety Disorder-7 (GAD-7), is a self-administered questionnaire developed by Robert L. Spitzer et al. with a sensitivity of 89% and specificity of 82% to assess the level of anxiety (18). It consists of seven items on a four-point Likert scale ranging from 0 to 3, in which 0 implies “not at all” and three implies “nearly every day.” The level of anxiety was categorized into four groups as minimal, mild, moderate, and severe based on scoring 0–4, 5–9, 10–14, and 15–21, respectively. Accordingly, minimal and mild were merged; “<10” was the absence of depression, and moderate and severe were merged for the presence of depression “≥10.” (18). The Cronbach's alpha for the anxiety was 0.8.

### Patient Health Questionnaire-9 (PHQ-9) for Depression

PHQ-9 developed by primary care evaluation of mental disorders (PRIME MD) was used to assess depression levels among participants. It consists of nine items on a four-point Likert scale ranging from 0 to 3, where 0 implies “not at all” and three implies “nearly every day.” The level of depression was categorized into five groups as minimal, mild, moderate, moderately severe, and severe based on scoring 0–4, 5–9, 10–14,15–19, and 20–27, respectively. Accordingly, minimal and mild were merged “<10;” and moderate, moderately severe, and severe was merged “≥10.” The scores (≥ 10) were used to determine the existence of depression (18). The Cronbach's alpha of the depression tool was 0.810.

### Inclusion and Exclusion Criteria

Participants who filled in the online survey form, above the age of 18 years old were included in the study. The decision for participation was completely voluntary. The students with no proper access to the internet facility and those who were unable to send the filled forms within the given deadline were excluded from the study. Also, the students with a history of exposure to Covid patients, Covid positive cases, currently taking mental care, and those working in health facilities were excluded from the study to minimize the confounding effect in the design of the study. It was assured by confirming with the respective participants. If they met the exclusion criteria, the google form was automatically sent over.

### Statistical Analysis

Data were collected and cleaned in Microsoft Excel. The data were imported to SPSS for further processing and analysis. Descriptive analysis such as frequency, percentage, mean and median was calculated. For inferential analysis, Chi squared test and logistic regression were performed. Anxiety and depression levels were assessed on the scoring. The association of scores was explored with socio-demographic characteristics, education-related factors, health-related factors, and technological-related factors.

## Results

The mean age of the respondents was 22.10 ± 2.928 years, ranging from 18 to 37 years, and more than half (55.7%) of the respondents were between the age of 21–25 years. More than three-fourths (83.1%) of the respondents were female and most (93.2%) of them followed Hinduism. More than two-thirds (67.7%) of the respondents were of upper caste groups and nearly half (48.2%) of them were living in municipalities. Most (87%) of the respondents were living in their own home (Table 1).

**Table 1:** Socio-demographic characteristics of the respondents (*n* = 409).

**Variables**	**Frequency (*n*)**	**Percentage (%)**
**Age**		
≤20 Years	138	33.7
21-25 Years	228	55.7
>26 Years	43	10.6
Mean ± SD (Min-Max) 22.1 ± 2.9 (18-37)		
**Sex**		
Male	69	16.9
Female	340	83.1
**Religion**		
Hinduism	381	93.2
Buddhism	20	4.9
Islam	4	1.0
Christianity	4	1.0
**Ethnicity**		
Dalit	10	2.4
Disadvantaged non-dalit terai caste	24	5.9
Religious minorities	3	0.7
Upper caste groups	277	67.7
Relatively advantaged janajati	95	23.2
**Place of residence**		
Rural municipality	31	7.6
Municipality	197	48.2
Sub-metropolitan	29	7.1
Metropolitan	152	37.2
**Living arrangement**		
Own home	359	87.8
Rent	50	12.2
**Living in status**		
With family	402	98.3
With friends	2	0.5
Without family	5	1.2

Most (41.3%) of the respondents were from Bagmati province and more than three-fourths (81.2%) of them had graduated. More than half (52.6%) of the respondents had a semester system and most (40.8%) of them were studying in the third and fourth semester of their 2nd year. Nearly half (47.9%) of the respondents were of public health stream and more than three fourths (81.7%) of them were attending virtual classes. Nearly three-fourths (71.9%) of the respondents had pending assignment/internal exams due to the lockdown and most (70.7%) of them had pending final exams, illustrated in Table 2.

**Table 2:** Education-related information of respondents.

**Variables**	**Frequency (*n*)**	**Percentage (%)**
**Provinces**		
Province one	33	8.1
Province two	25	6.1
Bagmati province	169	41.3
Gandaki province	140	34.2
Province five	29	7.1
Karnali province	13	3.2
**Level of education**		
Graduate	332	81.2
Post-graduate	77	18.8
**Education system**		
Semester	215	52.6
Annually	194	47.4
**Grade**		
First and second semester/first year	132	32.3
Third and fourth semester/second year	167	40.8
Fifth and sixth semester/third year	54	13.2
Seventh and eighth semester/fourth year	56	13.7
**Stream/discipline**		
Nursing	117	28.6
Public health	196	47.9
Pharmacy	31	7.6
MLT	51	12.5
Physiotherapy	14	3.4
**Presence of virtual class**		
Yes	334	81.7
No	75	18.3
**Frequency of virtual class/week (*****n*** **=** **334)**		
≤6 class/week	289	70.7
More than 6 class/week	45	11
Mean ± SD = 4.79 ± 3.48		
**Pending of assignment/internal exam**		
Yes	294	71.9
No	115	28.1
**Pending of final exam**		
Yes	289	70.7
No	120	29.3
**Time spent in internet for educational purpose**		
Don't spend	50	12.2
<4 h	307	75.1
More than 4 h	52	12.7
**Frequency of contact with teachers**		
No contact	113	27.6
Once times a day	169	41.3
More than one times	127	31.1

More than half (67.7%) of the respondents had changes in diet or food habits during the lockdown and had the chance to sleep in during the day. More than half (61.9%) of the respondents exercised or played sports and more than half (63.1%) of them did not play offline games. Details are presented in Table 3. More than three-fourths (83.1%) of the respondents had Wi-Fi as the source of internet, and more than half (62.6%) had strong internet signals. Almost half (49.4%) of the respondents used a mobile device to access the internet (Table 4). More than half (52.8%) of the respondents had minimal anxiety symptoms and more than half (57.9%) had minimal depressive symptoms. The prevalence of anxiety and depression was 15.7 and 10.7%, respectively. Details are illustrated in Table 5.

**Table 3:** Health-related information of respondents.

**Variables**	**Frequency (*n*)**	**Percentage (%)**
**Diet/change in food habit**		
Yes	277	67.7
No	132	32.3
**Rest/sleep**		
< average (≤6 h)	45	11
Normal (7–8 h)	216	52.8
More than average (≥9 h)	148	36.2
**Exercise/sports**		
Yes	253	61.9
No	156	38.3
**Play offline games**		
Yes	151	36.9
No	258	63.1

**Table 4:** Technological related information.

**Variables**	**Frequency (*n*)**	**Percentage (%)**
**Source of internet**		
Wi-Fi	340	83.1
Mobile data	48	11.7
Both	21	5.1
**Strength of internet**		
Strong	256	62.6
Weak	153	37.4
**Time spent in internet per day**		
<5 h	217	53.1
More than 5 h	192	46.9
**Device to access internet**		
Mobile and laptop	180	44.0
Mobile	202	49.4
Laptop	24	5.9
Desktop	3	0.7

**Table 5:** The rate of different severities of anxiety and depressive symptoms.

**Variables**	**Anxiety symptoms**		**Depressive symptoms**	
	**Frequency**	**Percentage (%)**	**Frequency**	**Percentage (%)**
Minimal	216	52.8	237	57.9
Mild	129	31.5	128	31.3
Moderate	42	10.3	33	8.1
Moderately	-	-	10	2.4
Severe	22	5.4	1	0.2

Final exam postponement was significantly associated with anxiety (*p* < 0.005) (Table 6). For students whose final exam was not postponed, the odds were 2.2 times higher than among the students whose final exam was postponed (OR = 2.2, 95%CI: 1.1–4.4). Province and hours spent on the internet per day for education were significantly associated with depression (p<0.05). With reference to students who spent less than the mean hours on the internet every day for education, the odds were 2.1 times higher among the students who spent more than mean hours on the internet per day for education (OR = 2.1, 95%CI: 1.1–4.2), as presented in Table 7.

**Table 6:** Bi-variate analysis between Anxiety and different variables (*n* = 409).

**Characteristics**	**Anxiety**		***χ^2^***	***P*-value**	**OR**	**CI**
	**Anxious**	**Non-anxious**				
**Educational level**						
Graduate	53 (16.0%)	279 (84%)	0.0.133	0.715	0.877	0.435–1.771
Post Graduate	11 (14.3%)	66 (85.7%)				
**Sex**						
Male	10 (14.5)	59 (85.5)	2.084	0.772	1.114	0.536–2.313
Female	54 (15.9)	286 (84.1)				
**Living arrangement**						
Home	54 (15)	305 (85)	0.817	0.366	1.412	0.666–2.992
Rent	10 (20)	40 (80)				
**Attending virtual class**						
Yes	50 (15)	284 (85)	0.634	0.426	0.767	0.399–1.475
No	14 (18.7)	61 (81.3)				
**Pending assignment/ internal exams**						
Yes	50 (17)	244 (83)	1.463	0.226	1.478	0.782–2.794
No	14 (12.2)	101 (87.8)				
**Final exam postpone**						
Yes	53 (18.3)	236 (81.7)	5.405	**0.020**	2.225	1.119–4.427
No	11 (9.2)	109 (90.8)				
**Exercise/play sports daily**						
Yes	36 (14.2)	217 (85.8)	1.011	0.315	0.758	0.442–1.301
No	28 (17.9)	128 (82.1)				
**Internet strength**						
Strong	36 (14.1)	220 (85.9)	1.303	0.254	1.369	0.797–2.350
Weak	28 (18.3)	125 (81.7)				
**Ability to operate virtual class application**						
Yes	53 (15.4)	292 (84.6)	0.136	0.712	0.875	0.429–1.783
No	11 (17.2)	53 (82.8)				
**Sleep per day**						
Less than average	9 (20)	36 (80)	0.761	0.643	-	-
Normal	32 (14.8)	184 (85.2)				
More than average	23 (15.5)	125 (84.5)				
**Hours of self-study per day**						
< =mean hour	31 (16.8)	154 (83.2)	0.315	0.575	0.858	0.503–1.464
>mean hour	33 (14.7)	191 (85.3)				
**Hours spent on the internet per day for education**						
< =mean hours	49 (15.1)	275 (84.9)	0.325	0.569	1.203	0.637–2.270
>mean hours	15 (17.6)	70 (82.4)				
**Frequency of virtual class per week**						
≤4.79 class	25 (16.7)	125 (83.3)	0.186	0.666	0.886	0.512–1.533
>4.79 class	39 (15.1)	220 (84.9)				
**Duration of virtual class per day**						
≤2.31 h	31 (14.2)	187 (85.8)	0.401	0.527	1.219	0.660–2.249
>2.31 h	20 (16.8)	99 (83.2)				
**Hours spent on the internet per day**						
≤5.85 h	28 (12.9)	189 (87.1)	2.638	0.104	1.558	0.910–2.666
>5.85 h	36 (18.8)	156 (81.2)				
**Hours spent on offline games per day**						
≤half hour	41 (16)	215 (84)	0.174	0.677	0.887	0.506–1.557
>half hour	22 (14.5)	130 (85.5)				

*Bold values represent statistical association*.

**Table 7:** Bi-variate analysis between depression and different variables (*n* = 409).

**Characteristics**	**Depression**		***χ^2^***	***P*-value**	**OR**	**CI**
	**Depressed**	**Non-depressed**				
**Educational level**						
Graduate	39 (11.7)	293 (88.3)	1.797	0.180	0.522	0.199–1.371
Post graduate	5 (6.5)	72 (93.5)				
**Provinces**						
Province 1	4 (12.1)	29 (87.9)	11.471	**0.031**		
Province 2	3 (12)	22 (88)				
Province 3	10 (5.9)	159 (94.1)				
Gandaki province	20 (14.3)	120 (85.7)				
Province 5	3 (10.3)	26 (89.7)				
Province 6	4 (30.8)	9 (69.2)				
**Sex**						
Male	6 (8.7)	63 (91.3)	0.368	0.544	1.321	0.536–3.259
Female	38 (11.2)	302 (88.8)				
**Attending virtual class**						
Yes	34 (10.2)	300 (89.8)	0.634	0.426	0.737	0.346–1.566
No	10 (13.3)	65 (86.7)				
**Pending assignment/ internal exams**						
Yes	36 (12.2)	258 (87.8)	2.408	0.121	1.866	0.840–4.147
No	8 (7)	107 (93)				
**Final exam postpone**						
Yes	30 (10.4)	259 (89.6)	0.146	0.702	0.877	0.447–1.720
No	14 (11.7)	106 (88.3)				
**Exercise/play sports daily**						
Yes	22 (50)	231 (91.3)	2.938	0.086	0.580	0.310–1.087
No	22 (14.1)	134 (85.9)				
**Ability to operate virtual class application**						
Yes	33 (9.6)	312 (90.4)	3.267	0.071	0.510	0.243–1.070
No	11 (17.2)	53 (82.8)				
**Sleep per day**						
Less than average	10 (22.2)	35 (77.8)	7.003	**0.043**	2.653	1.152–6.112
Normal	21 (9.7)	195 (90.3)			1	Ref
More than average	13 (8.8)	135 (91.2)			0.894	0.433–1.847
**Hours of self-study per day**						
≤mean hour	16 (8.6)	169 (91.4)	1.565	0.211	1.509	0.789–2.884
>mean hour	28 (12.5)	196 (87.5)				
**Hours spent on internet per day for education**						
>mean hours	15 (17.6)	70 (82.4)	5.304	**0.021**	2.180	1.109–4.284
≤mean hours	29 (9)	295 (91)			1	Ref
**Frequency of virtual class per week**						
≤4.79 class	16 (10.7)	134 (89.3)	0.002	0.964	1.015	0.530–1.945
>4.79 class	28 (10.8)	231 (89.2)				
**Hours spent on internet per day**						
≤5.85 h	21 (9.7)	196 (90.3)	0.562	0.453	1.270	0.679–2.376
>5.85 h	23 (12)	169 (88)				
**Hours spent on offline games per day**						
≤half hour	30 (11.7)	226 (88.3)	0.624	0.430	0.764	0.392–1.492
>half hour	14 (9.2)	138 (90.8)				

*Bold values represent statistical association*.

## Discussion

The study revealed that every one in 10 (10.7%) and nearly one in seven (15.7%) health sciences students who had internet access was suffering from moderate to severe depressive and anxiety symptoms. The findings showed a strong association between final exam postponement and anxiety. Sleeping during the day, hours spent on the internet, and coming from select provinces were associated with depression.

### Anxiety

The study showed that 52.8, 31.5, 10.3, and 5.4 percent of points were scored in minimal, mild, moderate, and severe anxiety, respectively. In contrast, slightly lower percent points in minimal (46.5%) and mild (18.7%), but higher of those in moderate (20.5%) and severe (14.3%) were observed in school adolescents from grades nine to 12 in Nepal (19). However, these adolescents were lower in age and might have less access to the internet. In addition, the current study findings contradict those of a study of Bangladeshis carried out by Islam S. et al. This study showed 18, 21, 47.3, and 13.8 percent of points for minimal, mild, moderate, and severe anxieties, respectively. It should also be noted that nearly all (98.2%) medical and allied health sciences students were Facebook users (20), showing that they have internet access.

This study reveals no association between engaging in exercise (*p* = 0.31, OR 0.7; 0.4–1.3) and anxiety, whereas the study done by Islam et.al shows a significant association between exercise and anxiety (*p* = 0.009, OR = 1.72; 95%, CI = 1.15–2.59). Respondents not engaging in physical exercise were 1.72 times more likely than the respondents taking physical exercise to have anxiety (21).

### Depression

Our study shows 57.9% minimal depression, 31.3% mild depression, 8.1% moderate, 2.4% moderately severe, and 0.2% severe depression. This contradicts findings by Islam et.al which shows 9.5% minimal depression, 11% mild depression, 50.2% moderate, 15.3% moderately severe, and 4% severe depression (21).

Our study shows that students who spend more time on the internet were 2.18 times more likely to be depressed than respondents spending less time on the internet (*p* = 0.021, OR = 2.1, CI = 1.1–4.2), whereas similar study done by Islam and et.al showed that the respondents using the internet <2 h/day were 0.53 times less likely than the respondents using the internet more than 4 h/day to be depressed (*p* = 0.033, OR = 0.53; 95% CI = 0.29–0.95).

In the present study, no statistically significant association was found between any of the socio-demographic variables, including education level, province, sex, religion, ethnicity, place of residence, staying partner, living arrangement, education system, and faculty of education. A previous study of Bangladeshi medical students also reported no significant relationship between socio-demographic variables and depression or anxiety (21). The present study only obtained significant associations with (i) place of residence /province and anxiety; (ii) sleep per day and depression; (iii) hours spent on the internet per day for education and depression; and, (iii) postpone of final exams and depression. A similar study conducted in Bangladesh also reported significant associations between lack of sleep satisfaction and depression, excessive daily internet use, and depression (21).

## Policy Implication

Based upon these insights into the psychological aspects of the pandemic, psycho-social counselors should be used by educational institutions, responsible for the health of the students. Mental health issues are prominent during pandemic situations, thus psycho-social aspects should be addressed at the initial stages. Educational institutions have to initiate policies and take actions that contribute to the promotion of mental health.

## Conclusion

Anxiety and depression in health science students showed a correlation with province, internet use for education, and postponement of exams. These correlations could be common among students of other fields as well. A large-scale study covering a wider geographical area and various fields of education is necessary to further evaluate the impact of COVID-19 on (health sciences) students. The integration of mental health programs both as an intervention and as part of the curriculum is critical to ensure the health of the students.

## Data Availability Statement

The raw data supporting the conclusions of this article will be made available by the authors, without undue reservation.

## Ethics Statement

The study received ethical approval from Nepal Health Research Council (No: 397/2020P) on 3rd June, 2020. Permission was also obtained from the selected colleges. All participants were fully informed regarding study objectives and written informed consent was obtained from participants by ticking off to agree to participate in research in Google form. Confidentiality of the data were fully maintained. All data were stored in the computer database which was accessible only to the researcher and was password protected.

## Author Contributions

RY, SM, DY, SB, HK, and EK: research conceptualization. RY, SB, SP, PP, RN, EK, and JP: supervised the data collection and writing-original draft. RY, SB, PP, RN, DY, HK, and CA: formal analysis. DY, SM, HK, CA, and RY: writing-review and editing. All authors read and approved the final manuscript.

## Conflict of Interest

The authors declare that the research was conducted in the absence of any commercial or financial relationships that could be construed as a potential conflict of interest.
